# Protocol for Japan–acute retinal necrosis (J-ARN) registry: A combined prospective and retrospective cohort study

**DOI:** 10.1371/journal.pone.0323822

**Published:** 2025-05-23

**Authors:** Yoshihiko Usui, Reiko Kinouchi, Satoko Nakano, Chiharu Iwahashi, Keitaro Hase, Kenji Nagata, Masahiro Sugimoto, Eiichi Hasegawa, Kinya Tsubota, Sentaro Kusuhara, Masato Akiyama, Kenji Kashiwagi, Atsunobu Takeda, Toshikatsu Kaburaki, Hiroshi Goto, Koh-Hei Sonoda

**Affiliations:** 1 Department of Ophthalmology, Tokyo Medical University, Shinjuku-ku, Tokyo, Japan; 2 Department of Ophthalmology, Asahikawa Medical University, Asahikawa, Hokkaido, Japan; 3 Department of Ophthalmology, Oita University, Yufu, Japan; 4 Department of Ophthalmology, Kindai University Faculty of Medicine, Osaka, Japan; 5 Department of Ophthalmology, Faculty of Medicine and Graduate School of Medicine, Hokkaido University, Sapporo, Japan; 6 Department of Ophthalmology, Kyoto Prefectural University of Medicine, Kyoto, Japan; 7 Institute of Medical Science, Tokyo Medical University, Tokyo, Japan; 8 Department of Ophthalmology, Graduate School of Medical Sciences, Kyushu University, Fukuoka, Japan; 9 Division of Ophthalmology, Graduate School of Medical Sciences, Kobe University, Kobe, Japan; 10 Department of Ophthalmology, Interdisciplinary Graduate School of Medicine, University of Yamanashi, Yamanashi, Japan; 11 Department of Ophthalmology, Jichi Medical University, Shimotsuke-City, Tochigi, Japan; PLOS: Public Library of Science, UNITED KINGDOM OF GREAT BRITAIN AND NORTHERN IRELAND

## Abstract

**Background:**

Acute retinal necrosis (ARN) is a rare but vision-threatening viral retinitis that can lead to severe visual impairment or blindness if not diagnosed and treated promptly. However, due to its rarity, there are limited large-scale data on the clinical characteristics, treatment approaches, and outcomes of ARN in Japan. A nationwide registry is needed to systematically collect data on ARN cases across Japan to improve understanding of this condition and optimize patient care. We have designed a national registry that collects data of patient characteristics, diagnosis, treatment, and visual outcome to generate evidence for the management of ARN.

**Methods and design:**

This research is a combined retrospective and prospective, multicenter cohort study of ARN in Japan from 1 April 2014–31 March 2029 (UMIN000056246). The registry has received Japan-wide approval from a national human research ethics committee. The following data will be collected: patient demographics, visual function at the initial visit and 6, 12, 24, and 36 months later, image data, diagnostic methods, virus analysis, indications and timing of vitrectomy, and complications. Customized software and platforms have been designed to permit data collection for a single baseline and multiple follow-up forms.

**Discussion:**

By analyzing the accumulated patient information, the results of this study will generate real-world evidence that will contribute to solve various important clinical issues in ARN. The results will be presented after data collection and analysis are completed.

## Introduction

Acute retinal necrosis (ARN) is a rare but severe infectious uveitis syndrome characterized by rapid necrosis of the retina, occlusive vasculopathy, and intense inflammatory responses within the eye. Delay in diagnosis and treatment can result in vision loss. This condition was first described in Japan in 1971 by Urayama et al. [1], and has since been recognized worldwide as a potentially sight-threatening ocular emergency. The causative agents of ARN are primarily members of the Herpesviridae family, including herpes simplex virus (HSV) types 1 and 2, and varicella-zoster virus (VZV). In Japan, real-time polymerase chain reaction (PCR) analysis of intraocular fluids is not covered by the universal health insurance system (public health insurance). However, identification of viral agents by performing PCR on intraocular fluid samples facilitates a definitive diagnosis of ARN [2–4]. Nevertheless, the diagnosis of ARN is primarily clinical, based on characteristic fundoscopic findings and supported by real-time PCR. A distinct feature of ARN is peripheral necrotic lesions that circumferentially progress to the posterior pole accompanied by occlusive vasculopathy, which is one of the diagnostic criteria of ARN [2]. The management of ARN poses several challenges, including difficulties in early diagnosis, variable responses to treatment, and a high rate of complications. The rapid progression of retinal necrosis and the potential of bilateral involvement necessitate prompt and aggressive intervention. However, the rarity of the condition and the lack of standardized treatment protocols complicate management. Moreover, the long-term prognosis remains guarded, with many patients experiencing recurrent episodes and permanent vision loss.

ARN is a relatively rare condition, with an annual incidence estimated at 0.63 cases per million population in the United Kingdom [5]. In Japan, ARN accounts for approximately 1.7% of all uveitis cases, underscoring its rarity but significant impact on those affected [6]. In Japan, where ARN has been recognized and studied extensively, the establishment of a nationwide registry represents a significant step toward improving patient care and advancing research in this challenging field. By consolidating data from multiple institutions, the registry will enable large-scale analyses that are not feasible in individual case reports or small case series. A report on the association between initial treatment regimen and retinal detachment rate and visual outcomes in patients with ARN in the American Academy of Ophthalmology IRIS^®^ Registry (Intelligent Research in Sight) has been published [7]. The insights gained from the nationwide registry in Japan will also be instrumental in addressing critical clinical questions, standardizing diagnostic and treatment protocols, and ultimately enhancing the quality of care for patients with ARN.

The primary objectives of the registry are to (1) characterize the clinical features, diagnostic approaches, virus type, and treatment patterns of ARN; (2) assess visual and anatomic outcomes in ARN patients treated with or without surgery; (3) identify visual prognostic factors associated with outcomes; and (4) compare the effectiveness of different treatment regimens. This information would provide valuable insights into ARN epidemiology, clinical features, treatment responses, and long-term outcomes. Such data are essential for developing evidence-based guidelines and optimizing management strategies. The registry would also facilitate collaborative research efforts facilitating the identification of prognostic factors and the evaluation of novel therapeutic approaches.

## Methods

### Study design and ethical considerations

The nationwide registry is named “Japan-acute retinal necrosis (J-ARN) registry”. The present research is a combined prospective and retrospective, multicenter, observational study of ARN in Japan from 1 April 2014–31 March 2029. The use of a combined retrospective and prospective design is due to the limited scope of the research that focuses on a rare and intractable disease with diverse treatment approaches. Recruiting a large number of participants is anticipated to be challenging.

**Fig 1:**
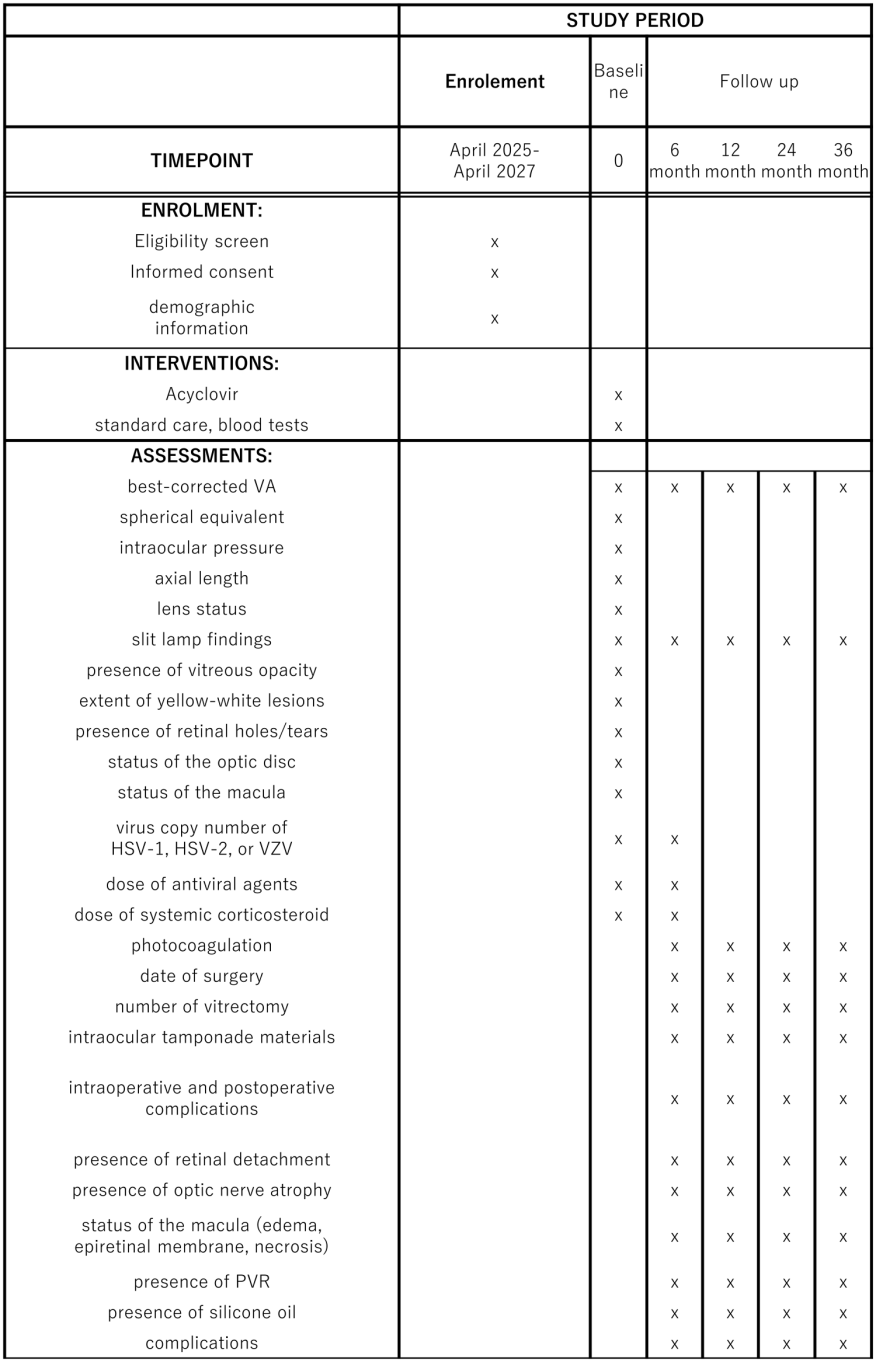
SPIRIT flow diagram for the schedule of enrolement, interventions, and assessments in our prospective study. Abbreviations: visual acuity (VA), herpes simplex virus (HSV), varicella zoster virus (VZV), proliferative vitreoretinopathy (PVR).

During the prospective recruitment phase, participating sites will enroll consecutive patients as they visit for routine follow-up, to reduce the risk of selection bias, and Fig 1 outlines timepoints of enrolment, interventions, and outcomes. Recruitment commenced on 1 April 2025. For the retrospective component, medical records of the prospectively recruited patients will be reviewed retrospectively back to 1 April 2014, to accurately determine the index date of the initial diagnosis of ARN. This hybrid approach aims to maximize the use of available data while ensuring robust and unbiased participant inclusion, despite the challenges associated with studying a rare disease.

The Oita University Hospital Clinical Research Supervision Center, Biomedical Research Ethics Review Board approved the study protocol (No. 2810-D28) on 23 May 2024, and the study is registered in the University Hospital Medical Information Network Clinical Trials Registry (UMIN000056246). The Tokyo Medical University Hospital Research Ethics Committee approved the study protocol (No. E2024-0071) on 1 November 2024. The study will adhere to the principles of the Declaration of Helsinki and the Ethical Guidelines for Life Science and Medical Research Involving Human Patients.

For the prospective component of the study, written informed consent will be obtained from all recruited participants, including consent for the PCR assay. However, written informed consent from patients will not be required for retrospective review of clinical charts, as the study design does not involve randomization. Instead, an opt-out approach will be employed in accordance with the Ethical Guidelines for Medical and Biological Research Involving Human Subjects.

### Sample size, inclusion and exclusion criteria

The goal of the study is to register a total of 500 eyes treated for ARN. The sample size was determined based on an estimation of number of eligible patients from a national uveitis survey in Japan [6]. Patients who meet the international diagnostic criteria for ARN and who have given written consent to participate in the prospective study, including intraocular fluid examination, will be eligible for registration [2,8]. Exclusion criteria are: (1) patient with retinal necrosis caused by pathogens other than HSV-1, HSV-2 or VZV, such as cytomegalovirus (CMV), (2) patient deemed unsuitable for the study by the investigator, and (3) patient in whom related diseases cannot be excluded despite meeting the inclusion criteria. In the case of exclusion from the study, clinical data until the date of consent acquisition will be registered, after which the study will be terminated.

### Diagnosis

Acute retinal necrosis is diagnosed based on the standard diagnostic criteria [2,8,9], including typical clinical presentation with peripheral necrotizing retinitis, and positive virologic tests using PCR analysis of intraocular fluids.

### Registered data

The following data will be collected (Table 1): height, weight, age, sex, laterality of ARN, medication history, history of shingles vaccination and herpetic diseases, drinking and smoking history, systemic disease, ocular disease, days from onset of ocular symptoms to first visit, subjective symptoms, best-corrected visual acuity, spherical equivalent, intraocular pressure, axial length, lens status, slit lamp finding (anterior chamber cells and flares, mutton-fat keratic precipitates), presence of vitreous opacity, fundus appearance (extent of yellowish white lesions), presence of retinal holes/tears, status of optic disc (swelling, redness, atrophy), status of the macula (edema, epiretinal membrane, necrosis), optical coherence tomography (OCT) data, treatment (antiviral agents, systemic corticosteroid, photocoagulation), date of surgery, surgical procedure, and complication.

**Table 1:** List of information collected for J-ARN registry.

Clinical data at initial visit	Treatment method, timing and adverse events	Events and viral copy number	Findings at 6, 12, 24, 36 months after initial visit
Height	Antiviral agents (intravitreal and/or intravenous and/or oral)	Maximum extension of yellow-white lesions	Best-corrected visual acuity
Weight	Systemic corticosteroids	Period until progression of yellow-white lesion stops	Slit lamp findings (AC cells, flare, mutton-fat KPs)
Age	Photocoagulation	Development of retinal detachment	Presence of retinal detachment
Sex	Surgery	Development of proliferative vitreoretinopathy	Presence of optic nerve atrophy
Race	Number of vitrectomy	Recurrence of ARN	Status of the macula (edema, epiretinal membrane, necrosis)
Systemic disease	Intraocular tamponade materials	Development of ARN in the contralateral eye	Presence of proliferative vitreoretinopathy
History of herpetic disease	Intraoperative and postoperative complications	Viral copy number measured during follow-up period	Presence of silicone oil
History of ocular disease			Complications
History of shingles vaccine			
Medication history			
Smoking history			
Days from onset of ocular symptoms to first visit			
Lesion laterality			
Subjective symptoms			
Best-corrected visual acuity			
Spherical equivalent			
Intraocular pressure			
Axial length			
Lens status			
Slit lamp findings (AC cells, flare, mutton-fat KPs)			
Presence of vitreous opacity			
Extent of yellow-white lesions			
Presence of retinal holes/tears			
Status of optic disc (swelling, redness, atrophy)			
Status of the macula (edema, epiretinal membrane, necrosis)			
Viral copy number of HSV-1, HSV-2 or VZV			

Abbreviations: AC, anterior chamber; ARN; acute retinal necrosis; KP, keratic precipitates; HSV, herpes simplex virus; VZV, varicella zoster virus

Decimal best-corrected visual acuity will be evaluated at the initial visit and at follow-up after 6, 12, 24, and 36 months. Decimal best-corrected visual acuity will be converted to logMAR units for statistical analysis. The collected data will be anonymized and registered online. The data center will be located within the hosting service of G-INGS Inc. (Fukuoka, Japan), ensuring maximum security for data storage and management. All data will be reviewed at least twice by the committee. The Committee will contact the participating institution for verification if any inconsistency or outlier is identified. If confirmation is not possible, the Committee will make the final decision.

A peripheral blood will be collected at the initial visit and thereafter as needed. Nonspecific inflammation tests such as erythrocyte sedimentation rate and blood count are sometimes included in the routine laboratory evaluation. The results of these tests are also essential for initiating therapy with anti-viral drugs and systemic corticosteroids.

### Safety

Because this is an observational study, participation does not involve any risk beyond those associated with the standard care for ARN in Japan. The registry does not contain any information that could identify patients. Each patient is assigned a unique number, and ophthalmologists are responsible for maintaining separate records of patient identities.

### Publication plan

The research findings derived from the registry will primarily be disseminated through peer-reviewed publications. These publications are anticipated to guide clinical practice and support the development of educational materials tailored for patients. Furthermore, the results may be shared at international uveitis conferences, aiming to foster global collaboration and knowledge exchange. Authorship of these publications will be attributed to the research group, which will include participating ophthalmologists and other research staff.

### Statistical analysis

The clinical profile as well as medical and surgical treatment options will be analyzed using descriptive statistics. Biostatisticians and information technology experts (M.A and M.S) will be involved in the planning, execution, and evaluation of the analyses. Multivariate analysis will be performed on changes in visual function over time and complications to identify influencing factors. Best-corrected visual acuity (BCVA) is converted to logarithm of the minimum angle of resolution (logMAR), and visual acuity worse than 0.01 is converted according to previous report [10] as follows: counting fingers, 2.6 logMAR; hand motions, 2.9 logMAR; light perception, 3.1 logMAR; no light perception, 3.4 logMAR. Data of continuous variables are expressed as mean and standard deviation or median and range, depending on normality of data distribution. Non-parametric data will be analyzed by Mann-Whitney U test or Kruskal-Wallis test, while parametric data will be analyzed by Student’s t-test or one-way ANOVA. Categorical variables will be expressed as total number and percentage, and the data will be analyzed by chi-squared test or Fisher’s exact test. The impact of clinical presentations, viral load, diagnostic methods, treatments, and complications will be estimated by odds ratios (ORs) with 95% confidence intervals (CIs). Survival analyses, especially Kaplan-Meier estimates, will be performed to evaluate visual prognosis, progression-free interval, and complications. Time-to-event data for 6, 12, 24, 36 month from the initial visit, the date of diagnosis, and/or vitrectomy were analyzed. A P value less than 0.05 will be considered statistically significant. Multivariate analyses (such as logistic regression analysis, cox proportional hazards regression, generalized estimating equations, and covariance structure analysis) will consider potential confounding variables related to treatment selection and outcomes.

There are primarily four types of machine learning algorithms: random forests, support vector machines, decision trees, and simple Bayesian classifiers. These algorithms are employed to integrate clinical data and image information for analysis purposes. Support vector machines utilize distinct kernels, and each method option and parameter is selected through cross-validation and bootstrap methods. This process aims to identify the combination that optimizes generalization. In contrast to deep learning, which necessitates a substantial number of cases, these machine learning methods ensure a certain level of accuracy even with a limited number of cases. The machine learning algorithms are designed to predict the outcomes of disease onset, diagnosis, treatment, visual function, and clinical decision-making.

### Discussion

Acute retinal necrosis (ARN) is a severe, rapidly progressive viral uveitis that leads to significant visual impairment or blindness if not treated promptly [11]. Despite advances in antiviral therapies and minimally invasive surgery, ARN remains a challenging disease to manage due to its aggressive nature and the complexity of its diagnosis and treatment. This article reports on the development of Japan’s national clinical registry, which will provide real-world data on the clinical course, management, and visual outcome of ARN. Using a multicenter approach, we aim to overcome the challenge posed by the low prevalence of ARN, which has been a major obstacle to advancing research and improving suboptimal outcomes. In addition to tracking visual outcomes associated with different treatment modalities, the registry will serve as a valuable resource for addressing key controversies surrounding the management of ARN. The mainstay for ARN treatment is systemic antiviral therapy, although which specific antiviral agent (e.g., acyclovir, ganciclovir or foscarnet) is superior to the others remains to be identified. In addition to systemic antiviral therapy, numerous studies have evaluated the therapeutic effects of adjunctive treatments such as intravitreal antiviral injections, systemic steroids, systemic immunosuppressants, prophylactic laser therapy, and prophylactic vitrectomy for the management of ARN and the prevention of retinal detachment [12–15]. The role of early and/or prophylactic vitrectomy in eyes without retinal detachment has been the subject of considerable debate. Some studies, such as that of Ishida et al. [16], have reported that prophylactic vitrectomy may be effective in preventing retinal detachment. However, other studies from Japan have shown that prophylactic vitrectomy does not improve visual outcome, because the outcome is largely determined by the extent of retinal and optic nerve damage at presentation, irrespective of the presence or absence of retinal detachment [17]. According to a meta-analysis of 265 eyes with ARN conducted by Fan et al. [18], prophylactic vitrectomy reduces the risk of retinal detachment. These findings suggest that although prophylactic vitrectomy may be beneficial in certain cases, it may not universally prevent retinal detachment in ARN patients due to the variability in efficacy. Therefore, the decision to perform prophylactic vitrectomy should be individualized, taking into account the extent of necrotic lesions and potential postoperative complications. The registry may provide insight into the effectiveness of different treatment regimens and their impact on visual outcome.

This registry has the potential to resolve the controversies by documenting patterns of clinical features, standardizing test procedures such as intraocular fluid sampling, and reviewing cases of misdiagnosis. Another important question that remains unanswered is the optimal approach to vitrectomy in patients with ARN—whether silicone oil tamponade, gas tamponade, scleral buckling, and photocoagulation should be used. The registry can address this issue by tracking visual outcomes after vitrectomy. The underlying challenge is the lack of robust evidence to determine the most effective treatment strategies for ARN patients, such as the type of treatment, whether to perform a vitrectomy alone or a combined procedure, and the timing of adjustment to the treatment regimen.

The second rationale for the proposed study is related to the fact that the number of viral copies in the intraocular fluids of patients with ARN significantly affects the visual prognosis of ARN.^19-21^ Calvo et al. [19] identified the presence of more than 5.0 × 10^6^ copies of VZV DNA in the aqueous humor of ARN patients as a poor prognostic factor. Another study [20] and our previous study [4] have reported that VZV-ARN is associated with a higher risk of retinal detachment. In general, VZV-ARN may be associated with worse visual prognosis compared to HSV-ARN [20], but a recent meta-analysis found no evidence to support a difference in visual outcome [21]. Therefore, a better understanding of virus types, viral copy number and clinical features may be important to improve visual prognosis of ARN and guide treatment options. In this regard, the proposed study provides an opportunity to expand current understanding of ARN.

Despite aggressive treatment, ARN carries a poor prognosis with high rates of retinal detachment and severe visual impairment [4]. A recent study reported that initial retinal detachment was detected in 2% of ARN patients, increasing to 47% during the follow-up period [13]. However, even after successful retinal reattachment, visual outcome often remains poor due to complications such as maculopathy and optic neuropathy [22]. The registry will track long-term outcomes including visual acuity, retinal detachment rate, complications, and recurrence, providing valuable data to guide future management strategies. Several studies have reported a significant association between the involved zone and the development of retinal detachment [13,23–26]. Furthermore, patients aged 50 years or younger diagnosed with ARN are more likely to develop retinal detachment and more severe ocular inflammation [27]. Several studies have investigated the prognostic factors that influence the clinical outcome of ARN. Choi et al. [28] identified poor initial visual acuity, necrotic retinitis in the central zone, and delayed retinal detachment onset as factors that significantly increase the risk of severe vision loss. Kim et al. [24] found that early diagnosis correlated strongly with better visual outcomes. Butler et al. [25] reported that zone 1 involvement and widespread retinal lesions were associated with worse visual prognosis. The proposed registry will evaluate the association between the development of retinal detachment and various clinical characteristics, and provide valuable data to guide future management strategies.

This registry study has several limitations. Because ARN is a rare disease, a combined retrospective and prospective study design is adopted to maximize data collection. For example, data extracted retrospectively from medical records may be incomplete due to the inherent limitations of retrospective data collection or insufficient data sources. Consequently, retrospective data may be more susceptible to selection bias than data collected prospectively. Furthermore, in this observational study, treatment methods are determined according to the clinical judgment of the ophthalmologists, making it impossible to control for variability. The number of patients included in the study will be small, and the results may not be generalizable to all regions of Japan. Clinical registries provide valuable opportunities to collect data in real-world clinical settings; however, there are inherent limitations to this research approach. As highlighted by Tan et al. [29] in their review of clinical registries in ophthalmology, limitations of clinical registries include potential selection bias due to the voluntary nature of participation by ophthalmologists. Additionally, missing information and typographical errors in data entry are noteworthy concerns. In future review of the registry, the scope of treatment descriptions could be broadened to include specifics such as the antiviral drugs used (e.g., acyclovir and foscarnet), dosages, and administration methods. This protocol will ensure that the field continues to explore innovative approaches. Accordingly, we expect the scope of data collected in the registry to expand as the project gains momentum.

## Conclusions

This study marks the first nationwide multicenter registry for ARN in Japan, and aims to gather detailed information on these patients. The registry will focus on the clinical characteristics, prevalence, treatment decisions, visual outcomes, and progression of ARN. It will also provide comprehensive insight into the etiology and pathogenesis of the disease. Ultimately, this effort aims to establish accurate visual prognoses for various medical and surgical treatments. The results of this registry will significantly enhance our understanding of the management of ARN. By identifying patients who are most likely to benefit from ARN-specific therapies or vitrectomy, the study can potentially improve treatment strategies and outcomes. In addition, this initiative sets the stage for future research to optimize treatment protocols and explore preventive measures against the progression of ARN.
